# Why is Clinical fMRI in a Resting State?

**DOI:** 10.3389/fneur.2019.00420

**Published:** 2019-04-24

**Authors:** Erin E. O'Connor, Thomas A. Zeffiro

**Affiliations:** Department of Diagnostic Radiology and Nuclear Medicine, University of Maryland Medical Center, Baltimore, MD, United States

**Keywords:** rs-fMRI, network, individuals, FDA, CPT code, ASFNR, survey

## Abstract

While resting state fMRI (rs-fMRI) has gained widespread application in neuroimaging clinical research, its penetration into clinical medicine has been more limited. We surveyed a neuroradiology professional group to ascertain their experience with rs-fMRI, identify perceived barriers to using rs-fMRI clinically and elicit suggestions about ways to facilitate its use in clinical practice. The electronic survey also collected information about demographics and work environment using Likert scales. We found that 90% of the respondents had adequate equipment to conduct rs-fMRI and 82% found rs-fMRI data easy to collect. Fifty-nine percent have used rs-fMRI in their past research and 72% reported plans to use rs-fMRI for research in the next year. Nevertheless, only 40% plan to use rs-fMRI in clinical practice in the next year and 82% agreed that their clinical fMRI use is largely confined to pre-surgical planning applications. To explore the reasons for the persistent low utilization of rs-fMRI in clinical applications, we identified barriers to clinical rs-fMRI use related to the availability of robust denoising procedures, single-subject analysis techniques, demonstration of functional connectivity map reliability, regulatory clearance, reimbursement, and neuroradiologist training opportunities. In conclusion, while rs-fMRI use in clinical neuroradiology practice is limited, enthusiasm appears to be quite high and there are several possible avenues in which further research and development may facilitate its penetration into clinical practice.

## Introduction

Techniques for quantifying spatial and temporal brain activity have developed rapidly since the first demonstrations that MRI could be used to measure modulations in blood oxygen level dependent (BOLD) tissue contrast (1). The observation that MRI could be used to monitor temporally correlated low-frequency activity fluctuations in spatially remote brain areas led to widespread use of resting state functional magnetic resonance imaging (rs-fMRI) to evaluate resting state network (RSN) properties. While BOLD-contrast is an indirect measure of neural activity, similar inter-regional coherent spontaneous neural activity correlations have been observed with electrophysiological techniques (2), suggesting that rs-fMRI networks can provide useful information about the macroscopic organization of neural processing systems. The methods and possible uses of rs-fMRI have recently been reviewed (3, 4).

Establishing that rs-fMRI can identify spontaneous brain activity patterns resembling those seen with tasks (5) has led to its widespread acceptance, and a rapid expansion in rs-fMRI publications. Nearly 10,000 rs-fMRI papers are currently listed in PubMed. The most rapidly developing type of functional connectivity research involves investigations of disease-related group differences in brain network structure, enabled by the relative simplicity of data collection from large samples. As a result, atypical resting-state connectivity has been demonstrated in a wide range of neuropsychiatric disorders, including epilepsy, schizophrenia, attention deficit hyperactivity disorder, Alzheimer's disease, stroke, and traumatic brain injury.

rs-fMRI has several advantages over task-fMRI in clinical contexts. First, data acquisition is less complex. Second, if mapping multiple neural systems is needed, rs-fMRI can identify them simultaneously, saving time. Finally, rs-fMRI can be performed in individuals unable to cooperate for fMRI tasks, such as young, sedated, paralyzed, comatose, aphasic, or cognitively impaired patients. In addition to its utility in detecting changes in group network properties, rs-fMRI can also be used to detect individual differences (6–10).

Although the first reports of rs-fMRI clinical applications appeared 10 years ago (11), rs-fMRI use in clinical neuroradiology practice remains in a nascent stage, limited mainly to pre-surgical planning (4, 12) and is typically performed in conjunction with task-fMRI. Given the rapid rise and widespread use of rs-fMRI in neuroimaging clinical research, it might be expected that rs-fMRI would already be widely used in clinical practice, particularly in academic centers. Nevertheless, this is not the case and the reasons for the relatively weak penetration of rs-fMRI methods into neuroradiology practice are not entirely clear. To determine attitudes toward the use of rs-fMRI use in neuroradiology research and practice, we recently queried the American Society for Functional Neuroradiology (ASFNR) membership. In this article we will discuss the results of this survey, covering opinions about the current state of rs-fMRI acquisition, analysis, and interpretation methods. We then address existing barriers to using rs-fMRI in clinical practice and propose possible solutions, presenting examples of typical group and individual subject rs-fMRI analyses using public domain data.

## Methods

After obtaining a human subjects research exemption, an invitation to participate in a 20 item electronic survey was sent to ASFNR members to collect information concerning their use of rs-fMRI in clinical research and practice, demographics, and work environment. Responses were collected using 5-point Likert items and deidentified prior to analysis.

Because a majority of respondents expressed concerns that substantial analysis and interpretation problems need to be solved before rs-fMRI can be widely used in clinical practice, we next explored examples of typical rs-fMRI analysis variations using the publicly available NYU CSC TRT dataset (www.nitrc.org/projects/nyu_trt), processed using the CONN Toolbox (13), a popular open-source rs-fMRI analysis program (www.nitrc.org/projects/conn). In one example, we explored the serial influence of time series preprocessing algorithms on language network detection using an inferior frontal gyrus ROI. Effects of applying global signal regression, incorporating head motion estimates, using anatomical CompCorr, and outlier elimination were examined in a group level analysis of 25 healthy participants. Next, we explored the effects of denoising on single participant data. The exercise revealed large effects that processing variations can have on the detection of domain-specific maps at the group or single-subject level. These results are presented in the discussion of existing barriers related to increasing rs-fMRI use in clinical practice.

## Results

The response rate was 24% (71/294). Of these, the majority were involved in both clinical and research activities. Twenty-one percent were female. Eighty-seven percent held MD, MBBS, or MD PhD degrees; the others were PhDs. Only two of the respondents were exclusively involved in research. The median time since training was 12 years.

Ninety-two percent of the ASFNR respondents reported having adequate MRI equipment to conduct rs-fMRI and 82% indicated that rs-fMRI was relatively easy to collect.

Eighty percent reported using task-fMRI and 59% reported using rs-fMRI in their past research. Seventy-two percent reported plans to use rs-fMRI for research in the next year. Yet, only 40% agreed, or strongly agreed, that they would use rs-fMRI in clinical practice in the next year. Eighty-two percent of respondents agreed or strongly agreed that task-fMRI and rs-fMRI clinical use are largely confined to pre-surgical planning, mentioning seizure focus detection as other promising application. Thirty-two percent agreed, or strongly agreed, that rs-fMRI is currently useful in pre-surgical planning and 68% agreed, or strongly agreed, that it will be useful in future surgical planning (Supplement Table 1).

**Figure 1:**
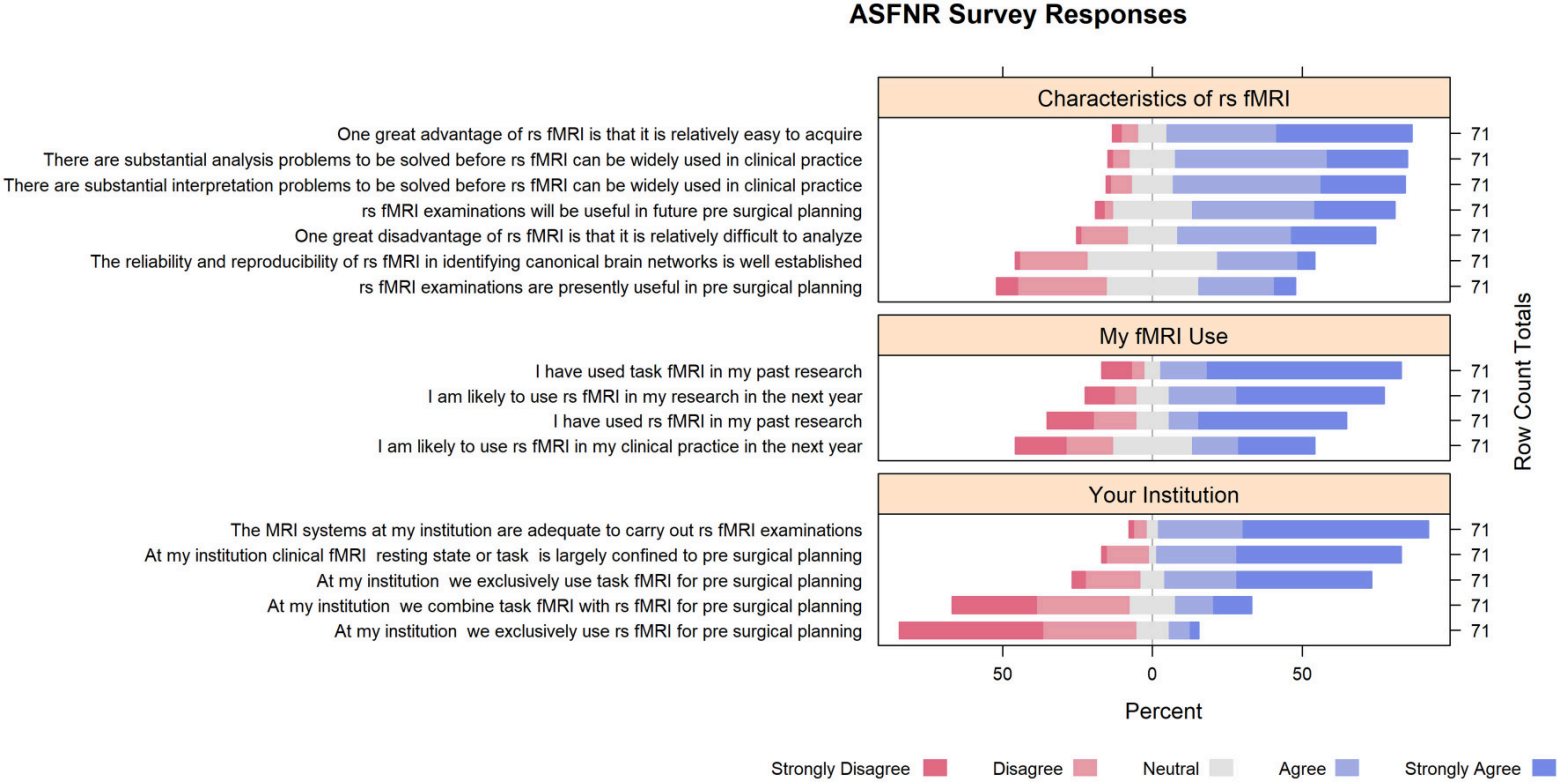
Survey responses.

While respondents expressed strong interest in rs-fMRI clinical applications, they expressed concerns that may explain its lack of penetration into clinical practice. For example, 66% agreed, or strongly agreed, that rs-fMRI data are difficult to analyze. Twenty-four percent expressed concern about the reliability and reproducibility of rs-fMRI in identifying canonical brain networks. Seventy-seven percent agreed, or strongly agreed, that there are substantial analysis problems to be solved before rs-fMRI can be widely used in clinical practice. In addition, 77% agreed, or strongly agreed, that there are substantial interpretation problems to be solved before rs-fMRI can be widely used in clinical practice (Figure 1).

## Discussion

In summary, while most respondents had experience with fMRI in both clinical and research contexts, have adequate MRI systems at their institutions and are relatively enthusiastic about incorporating rs-fMRI into clinical protocols, a number of concerns appear to be slowing the translation of rs-fMRI from research to practice. Some barriers to rs-fMRI implementation in clinical practice, and possible ways to circumvent them, are addressed below.

### Barrier 1: Precision Medicine Agenda

Functional MRI used in research settings typically averages participant data in order to detect differences in regional task effects between clinical and healthy groups. In clinical medicine, however, diagnostic inferences and treatment recommendations are made for single cases.

As most publications describe acquisition and analysis methods optimized to detect between-group effects, better methods to characterize rs-fMRI maps in individuals are needed. Acquisition technology advances, such as higher magnetic field strength, multi-channel coils, and faster image acquisition have led to substantial sensitivity improvements, making the study of individual resting state networks possible (14).

One simple way to improve network detection sensitivity is to lengthen scan time. While some canonical RSNs, such as the default mode or sensorimotor networks, can be reliably detected at the group level using 5–6 min scans, longer sampling times, on the order of 12–30 min, can substantially improve detection of networks exhibiting lower average connectivity (15, 16). Since rs-fMRI data is dominated by physiological noise, longer sampling times with short TRs allow more effective physiological denoising and more sensitive neural signal detection. While most analysis techniques assume static connectivity effects between pairs of network nodes, dynamic connectivity estimates can benefit even more from longer sampling times. Dynamic connectivity analysis, while relatively new to rs-fMRI, holds promise in providing quantitative estimates of time-varying connection phenomena that may be altered in brain disease (17).

Variance in intrinsic connectivity contributed by cognitive state and mood, rather than disease effects, may be responsible for individual network structure variation (18). Nevertheless, moderate-to-high test-retest reliability of rs-fMRI indices challenges these concerns (19). In addition, longer sampling times, as discussed above, can facilitate detection of individual static network structure in the face of moderate dynamic variations in connectivity.

While rs-fMRI is currently being used for preoperative planning in a few centers (20), other clinical applications are not as common. High within-subject reproducibility of RSNs suggests that they might serve as biomarkers for monitoring disease progression in individual patients (21).

Finally, tools comparing individual to group maps are needed. Structural templates based on normative data sets that take into account age, sex, magnetic field strength, and data quality have been developed (22). Standardizing rs-fMRI acquisition protocols, then collecting normative comparative data, would greatly facilitate rs-fMRI clinical use by allowing comparison of individuals to age, sex, and IQ adjusted norms. For example, a clinically relevant target, the left hemisphere language network, when identified using a left inferior gyrus ROI, exhibits substantial between-subject variability, even when averaging across three collection sessions (Supplement Figure 1). Of greater concern is the fact that the majority of patients referred for pre-surgical mapping have space occupying lesions that distort both local and global anatomy, making mapping to standard anatomical spaces difficult or impossible using conventional spatial normalization techniques. Moreover, slowly growing tumors may dynamically alter inter-regional connectivity, making comparisons to functional group maps derived from healthy participants difficult to interpret. In pre-surgical planning, precisely determining the details of how an individual patient's functional anatomy differs from a typical spatial distribution may be important in determining treatment recommendations.

### Barrier 2: Diversity of Measures

Numerous methods can characterize regional intrinsic connectivity, including ROI->ROI correlations, ROI->voxel correlations, independent component analysis (ICA) of canonical networks, dynamic functional connectivity analysis, and graph theory analysis [see (3, 23) for recent reviews]. These different connectivity modeling techniques may measure fundamentally different aspects of inter-regional coupling.

It is also unclear which connectivity measures are sensitive to specific pathologies and therefore are most appropriate to particular clinical questions. ROI->ROI analysis is useful for identifying low spatial resolution network properties and is computationally efficient due to the low number of correlations computed. ROI->voxel approaches reveal more spatial detail, at the cost of greatly increased calculation time. Voxel->voxel methods, such as ICA, are the most computationally demanding, but do not require a priori anatomical assumptions, and thus may be better suited for exploratory studies of network structure (14). In addition, techniques for ICA network identification have not been standardized and are quite sensitive to specification of the maximum number of identified components. Increasing the maximum number can cause large networks to split into smaller subsets. A major limitation of network analysis methods based on graph theory metrics is that group sizes larger than 40–50 are required to obtain stable estimates of network properties using short acquisition protocols, making them difficult to use in characterizing individual patients (24). Nevertheless, novel indices, like the hub disruption index, may be useful in characterizing an individual's relationship to a group (25). For all of these techniques, compensating for anatomical distortion from space occupying lesions presents a substantial analytical challenge.

### Barrier 3: Reliability and Reproducibility

Recently, there has been growing concern about the reliability and reproducibility of biomedical research (26). Our survey demonstrates that the neuroradiology community shares this concern with respect to rs-fMRI.

Identifying reliable and reproducible canonical brain networks has received great attention in the rs-fMRI literature, with studies showing reproducible networks in both adults and children (27, 28). Yet, the neuroradiology community remains uncertain about how these findings translate to individual patients. More individual participant test-retest studies may be needed to address this area of uncertainty.

Large test-retest data sets, focusing on rs-fMRI from over 36 laboratories around the world, have been made publicly available by the Consortium for Reliability and Reproducibility (CoRR) through the International Data-sharing Neuroimaging Initiative (29). The individual scans composing the large aggregate dataset have been collected using different acquisition parameters and experimental designs, allowing investigators to assess rs-fMRI reliability and reproducibility. In addition, the impact of commonly encountered artifacts, such as motion, on inter-individual variation can be explored (29). Publicly available datasets from the NIH supported Human Connectome Project (http://humanconnectome.org) are also being used to evaluate the reliability of rs-fMRI and functional connectivity summary measures (30).

In addition, there have not yet been any large scale validation studies to determine if the cognitive domains commonly mapped using intraoperative cortical stimulation can be identified using rs-fMRI. Most rs-fMRI validation studies compare to task-fMRI results, which are expected to have better specificity for specific functions, making simple comparisons difficult. Comparisons between cortical stimulation and other functional imaging modalities have previously shown good between modality correspondence (31), suggesting that this strategy may be useful.

### Barrier 4: rs-fMRI Analysis Issues

While a majority of survey respondents indicated that rs-fMRI data are relatively easy to collect, the majority also believed that rs-fMRI data are relatively difficult to process.

Resting state data analysis can be time intensive and, therefore, not always feasible during a typical demanding day on clinical service. Automatic transfer of images to a clinical image archiving system, followed by automated analysis, could facilitate clinical workflows. One popular analysis program, the CONN Toolbox (13), while well suited for automated analysis of group rs-fMRI data, has limited options for single subject statistical analysis. Nevertheless, a CONN Toolbox script optimized for clinical use and running on a typical laboratory computer requires 10–15 min to process data from a single subject, in addition to the time required to transfer images from PACS. Other toolboxes designed for clinical practitioners, such as CLINICA (32), are not yet widely used, but do hold promise for single subject analysis.

Hemodynamic signal artifacts resulting from physiological noise, including head motion, cardiac pulsation, and respiratory effects can severely compromise efforts to detect regional modulations in neural activity.

Participant head motion is particularly problematic, as it can bias estimated activity correlations between regions. Visual examination of a participant's scan immediately after completion, using a movie loop, allows a clinician to repeat scans when excessive head motion is detected. Nevertheless, even small inter-scan head movements (<0.5 mm) can bias correlation estimates, influencing between-group effect estimates (33). For this reason, motion correction using rigid body realignment is an obligate part of the rs-fMRI preprocessing pipeline, followed by inclusion of motion estimates in subsequent single-subject statistical modeling (34).

Even images from cooperative patients will have physiologic confounds that need to be addressed. Cardiac pulsation and respiration can cause spurious connectivity patterns (35). Band-pass filtering to remove fluctuations outside the frequency range of interest mitigates cardiac and respiratory effects and does not require external physiological recordings. Filtering frequencies lower than ~0.01 Hz and >~0.2 Hz, reduces the effects of non-neuronal physiologic processes (36).

**Figure 2:**
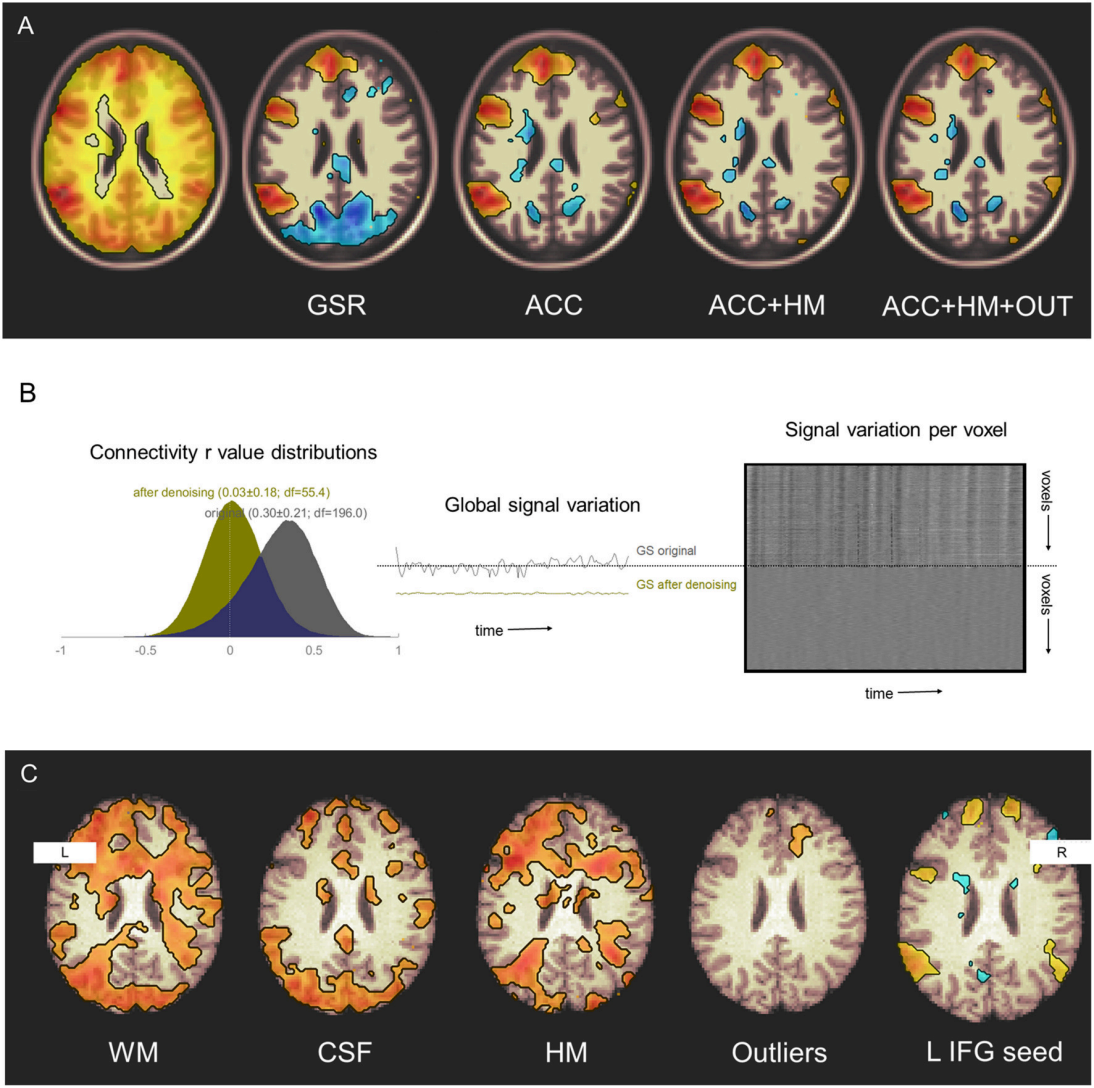
Denoising effects on functional connectivity estimates. **(A)** Additive effects of denoising sources on detection of seed connectivity in a group of 25 healthy participants studied during three sessions. GSR, global signal regression; ACC, anatomical CompCorr; HM, head motion estimates; OUT, head motion and global intensity outliers. Display threshold *p* < 0.001. Original data from NYU CSC TRT: subjects 1–25, sessions 1–3. **(B)** Denoising reduces structured noise in individuals. Left–Connectivity values histograms in a single healthy participant before (gray) and after (yellow) denoising including WM signal, CSF signal, estimated head motion, and outlier removal. Middle–Global signal variation before and after denoising. Right–carpet plot of voxel signal variation before (top) and after (bottom) denoising. Original data from NYU CSC TRT: subject 16, session 1. **(C)** Denoising increases sensitivity to, and specificity for, the language network. Effects of including denoising sources on detection of left inferior frontal gyrus seed connectivity are seen in a single participant. WM, white matter; CSF, cerebrospinal fluid; HM, head motion estimates; Outliers, head motion and global intensity outliers. Display threshold *r* = 0.4. Original data from NYCSC TRT: subject 16, session 1.

Global signal regression (20) is another method sometimes used for physiologic noise reduction (37). GSR uses a denoising covariate that contains information from both physiological noise and neural signal. Its re-centers the mean of the inter-regional correlation distribution, so that some positive correlations appear to be negative. Its use may therefore confound attempts to distinguish sets of regions whose activity are either positively or negatively associated (38). For this reason, noise reduction techniques like anatomical CompCorr, that exclude the cortical signal from the denoising procedure, may be preferred in most circumstances (13) (Figure 2).

Systemic carbon dioxide (CO_2_) fluctuations alter BOLD-contrast and contribute to respiratory induced signal variation (39). To reduce CO_2_ fluctuation effects, end-tidal CO_2_ can be measured with a face-mask or nasal cannula and the measurements incorporated into the denoising pipeline (39).

Temporal signal-to-noise ratio (tSNR), the ratio of the mean signal over its temporal standard deviation (SD), reflects the ability to detect BOLD-contrast signal changes (40), and thus can be used in quality assurance. More recently, the Physiological Contributions in Spontaneous Oscillations index has been proposed as a more sensitive measure of functional connectivity strength (41).

These denoising techniques are not only effective at the group level (Figures 2A,B), but also can improve sensitivity and specificity for detecting networks at the individual level (Figure 2C).

In summary, the inter-regional associations estimated with rs-fMRI may be relatively weak compared to the customary task-fMRI effects, often being masked by physiological noise. The reproducibility of the two modalities may also differ. Varying acquisition and processing parameters can profoundly affect detection sensitivity (42) and there is ongoing debate regarding the role of GSR in pre-processing (43–45). Further, different data analysis families such as ROI-based correlation analysis, independent component analysis (ICA) detection of canonical networks, and graph theory metrics used to quantify local and global network properties, are likely to be sensitive to very different aspects of inter-regional functional connectivity (3).

To allow readers to reproduce the denoising pipeline variations shown in Figure 2, links are provided to scripts that preprocess and model the NYU CSC TRT dataset (www.neurometrika.org/tutorials/fc-denoising).

### Barrier 5: User Training

Traditionally, diagnostic radiology has been primarily an anatomical medical specialty. Functional MRI acquisition and interpretation is more physiological and statistical in nature and may therefore may require somewhat different training.

While many academic programs briefly expose trainees to the principles of functional MRI, it is presently not part of the standard curriculum in diagnostic radiology residency or neuroradiology fellowship programs in the U.S. More training in software systems for rs-fMRI analysis will facilitate clinical practice implementation. Relevant curricular offerings in systems neuroscience and statistical modeling could help trainees gain a deeper understanding of the origins of instrumental and physiological noise in rs-fMRI data and thereby optimize their data acquisition, analysis, and interpretation efforts.

### Barrier 6: Standardization, Regulatory, and Financial Issues

The lack of standardization of rs-fMRI acquisition and analysis methods may reflect a lack of consensus regarding the best approach to maximize inter-individual signal variability while concomitantly minimizing intra-subject measure variability (46). As task-fMRI analysis methods are relatively mature compared to their rs-fMRI counterparts, more vigorous engagement of professional societies with the rs-fMRI research community will promote achieving agreement concerning rs-fMRI analysis standards.

Of great importance from a practical viewpoint, there is currently no FDA-cleared software for rs-fMRI analysis on MRI consoles. Obtaining FDA marketing authorization for rs-fMRI clinical use will require validating its intended use as a “tool type” device and more clearly determining what the statistical information derived from rs-fMRI means for patient diagnosis and treatment. Overcoming these hurdles will require a concerted effort from the interested academic and commercial parties. MRI system vendors could have a major role in these activities, working with academic investigators to develop software tools and techniques in accordance with standard medical device development practices, thereby speeding the transition from research to practice.

Acquiring the expertise needed for rs-fMRI acquisition, analysis, and interpretation requires a substantial time commitment. Busy clinicians may be more motivated to obtain such training, and their associated hospitals be more willing to support them, if rs-fMRI had an associated Current Procedural Terminology (CPT) code. Before this can happen, however, rs-fMRI protocols must be standardized by neuroradiologists. Task-fMRI received a CPT code in the U.S. after relative standardization of the processing and analysis techniques. Societies such as the RSNA, ASNR, and ASFNR may be more likely to pursue the process of obtaining an rs-fMRI CPT code after clinical validation and standardization has been achieved.

Even after standardization and regulatory hurdles are overcome, it will be necessary to identify the clinical applications for which rs-fMRI can provide useful information to referring physicians from neurosurgery, neurology and psychiatry. For example, preoperative mapping of motor and language brain function, the most common clinical application of fMRI and rs-fMRI, has been widely integrated into pre-surgical planning protocols in academic centers (32, 47). While resting-state pre-surgical maps can reliably identify sensorimotor function (12, 48, 49), larger scale validation studies are still needed, and solving problems related to substantial subject level variability remains for language mapping (50, 51) (Supplement Figure 1). Individual subject level reliability still needs to be addressed with large studies before clinical services will routinely request rs-fMRI for clinical practice.

## Limitations

Our study has limitations. First, our response rate was 24% of the ASFNR membership and respondents may have tended to be more enthusiastic about using rs-fMRI in their research and clinical practice than non-respondents. Second, surveys were only sent to the ASFNR membership and thus non-member neuroradiologists who use rs-fMRI were not sampled. Third, for practical reasons, our survey was confined to members of an American professional organization. It will be of interest to survey a broader and more international sample of the neuroimaging community to assess the generality of our findings and interpretations.

## Conclusions

Despite some perceived impediments to expanding clinical rs-fMRI use, neuroradiologists were generally enthusiastic about rs-fMRI in research and clinical applications, believing that their current workplace MRI systems are suitable for rs-fMRI acquisition. Many of the concerns associated with using rs-fMRI in clinical contexts are related to: (1) developing better methods for minimizing physiological noise effects, (2) improving methods for detecting the spatial characteristics of clinically-relevant brain processing systems in individual patients, and (3) overcoming remaining standardization, training, and regulatory hurdles.

### Author Contributions

EO and TZ contributed to study design, data collection and analysis, and manuscript preparation.

### Conflict of Interest Statement

The authors declare that the research was conducted in the absence of any commercial or financial relationships that could be construed as a potential conflict of interest.

## Abbreviations

BOLD: blood oxygen level dependence
rs-fMRI: resting state functional magnetic resonance imaging
ASFNR: American Society of Functional Neuroradiology
RSNs: resting state networks
ICA: independent component analysis
ROI: region of interest.
