# Supplementary material for: Why is Clinical fMRI in a Resting State?
Source: Front Neurol. 2019 Apr 24;10:420. doi: 10.3389/fneur.2019.00420 (PMC6491723; doi:10.3389/fneur.2019.00420)
Supplement: Supplementary file 1 [file Table_1.DOCX]

Supplement Figure 1 Twelve single participant surface displays of left IFG connectivity after denoising with anatomical CompCorr, motion regression and outlier regression. Display threshold r=0.25. The left hemisphere language network exhibits considerable spatial variability among participants. Data from NYU_TRT subject 1-12 session 1-3


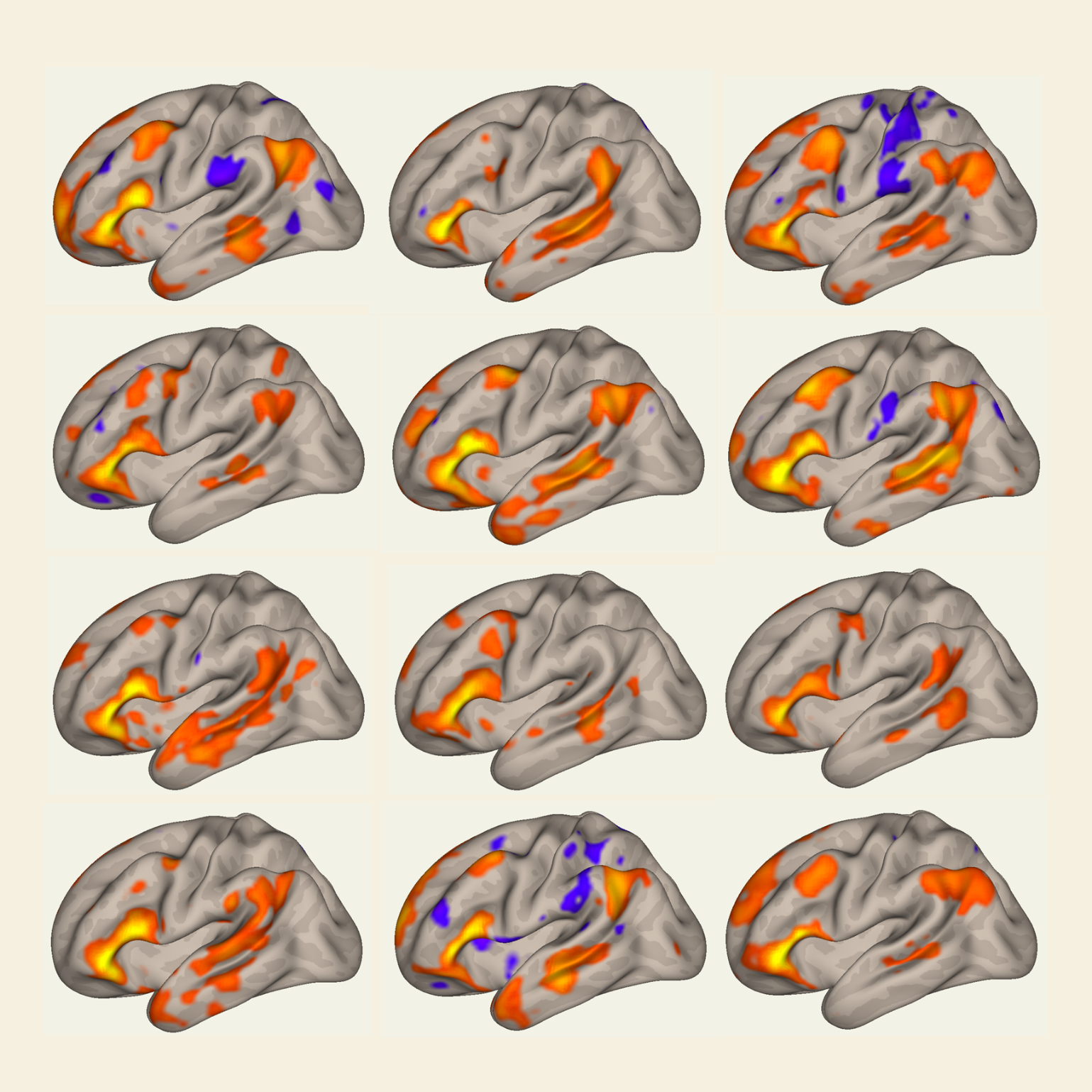


Supplement Table 1: Survey results

| *Category* | *Question* | *Strongly Disagree* | *Disagree* | *Neutral* | *Agree* | *Strongly Agree* |
| --- | --- | --- | --- | --- | --- | --- |
| Characteristics of rs-fMRI | One great advantage of rs-fMRI is that it is relatively easy to acquire | 2 | 4 | 7 | 26 | 32 |
| Characteristics of rs-fMRI | One great disadvantage of rs-fMRI is that it is relatively difficult to analyze | 1 | 11 | 12 | 27 | 20 |
| Characteristics of rs-fMRI | rs-fMRI examinations are presently useful in pre surgical planning | 5 | 21 | 22 | 18 | 5 |
| Characteristics of rs-fMRI | rs-fMRI examinations will be useful in future pre surgical planning | 2 | 2 | 19 | 29 | 19 |
| Characteristics of rs-fMRI | The reliability and reproducibility of rs-fMRI in identifying canonical brain networks is well established | 1 | 16 | 31 | 19 | 4 |
| Characteristics of rs-fMRI | There are substantial analysis problems to be solved before rs-fMRI can be widely used in clinical practice | 1 | 4 | 11 | 36 | 19 |
| Characteristics of rs-fMRI | There are substantial interpretation problems to be solved before rs-fMRI can be widely used in clinical practice | 1 | 5 | 10 | 35 | 20 |
| My fMRI Use | I am likely to use rs-fMRI in my clinical practice in the next year | 12 | 11 | 19 | 11 | 18 |
| My fMRI Use | I am likely to use rs-fMRI in my research in the next year | 7 | 5 | 8 | 16 | 35 |
| My fMRI Use | I have used rs-fMRI in my past research | 11 | 10 | 8 | 7 | 35 |
| My fMRI Use | I have used task fMRI in my past research | 7 | 3 | 4 | 11 | 46 |
| Your Institution | At my institution we combine task fMRI with rs-fMRI for pre-surgical planning | 20 | 22 | 11 | 9 | 9 |
| Your Institution | At my institution we exclusively use rs-fMRI for pre surgical planning | 34 | 22 | 8 | 5 | 2 |
| Your Institution | At my institution we exclusively use task fMRI for pre-surgical planning | 3 | 13 | 6 | 17 | 32 |
| Your Institution | At my institution clinical fMRI resting state or task is largely confined to pre-surgical planning | 1 | 10 | 2 | 19 | 39 |
| Your Institution | The MRI systems at my institution are adequate to carry out rs-fMRI examinations | 1 | 3 | 3 | 20 | 44 |
